# Higher food prices can reduce poverty and stimulate growth in food production

**DOI:** 10.1038/s43016-023-00816-8

**Published:** 2023-08-10

**Authors:** Derek Headey, Kalle Hirvonen

**Affiliations:** 1 International Food Policy Research Institute (IFPRI), Colombo, Sri Lanka; 2 United Nations University World Institute for Development Economics Research (UNU-WIDER), Helsinki, Finland

**Keywords:** Economics, Development studies, Developing world

## Abstract

Food prices spiked sharply in 2007–2008, in 2010–2011 and again in 2021–2022. However, the impacts of these spikes on poverty remain controversial; while food is a large expense for the poor, many poor people also earn income from producing or marketing food, and higher prices should incentivize greater food production. Short-run simulation models assume away production and wage adjustments, and probably underestimate food production by the poor. Here we analyse annual data on poverty rates, real food price changes and food production growth for 33 middle-income countries from 2000 to 2019 based on World Bank poverty measures. Panel regressions show that year-on-year increases in the real price of food predict reductions in the US$3.20-per-day poverty headcount, except in more urban or non-agrarian countries. A plausible explanation is that rising food prices stimulate short-run agricultural supply responses that induce increased demand for unskilled labour and increases in wages.

## Abstract (web summary)

This study presents a panel-data analysis of 33 middle-income countries over 2000–2019 and reveals that increases in real food prices have reduced poverty in more agrarian economies but seemed to have little or no impact on aggregate poverty in more urbanized economies. Short-run agricultural supply responses emerge as a key mechanism by which higher food prices could reduce poverty in specific contexts.

## Main

**Fig. 1:**
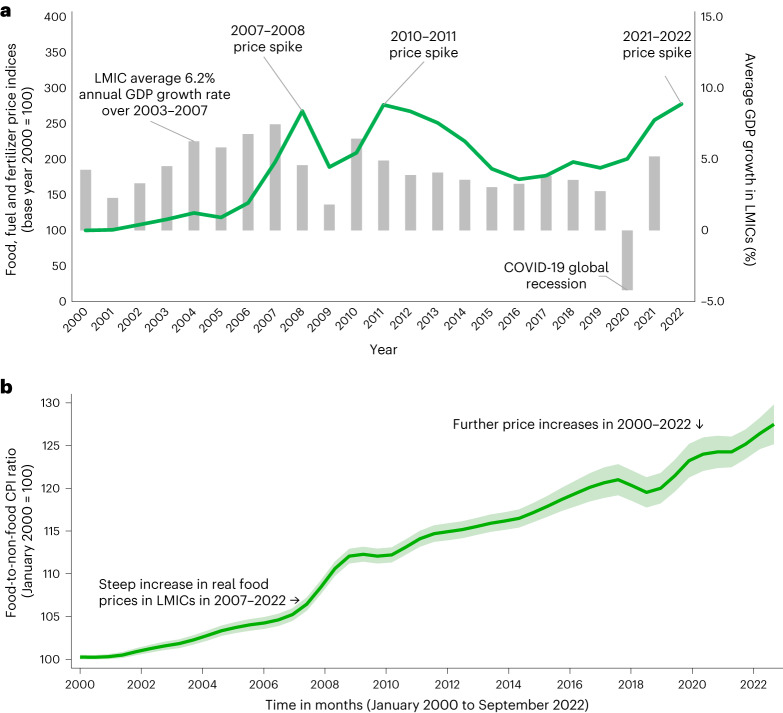
Trends in the international food price index and economic growth in low- and middle-income countries (a) and trends in the domestic real food price index from 2000 to 2022 in low- and middle-income countries (b). **a**,**b**, Panel **a** shows trends in the FAO cereal price index and World Bank data on growth in GDP per capita. The price indices all refer to price data from major agricultural exporters. Panel **b** shows a local polynomial regression of the food CPI-to-total CPI ratio sourced from the FAO against time in months from January 2000 to September 2022 for 92 low- and middle-income countries, with 25,080 observations. The solid green line represents the predicted index value of the real price of food across all 92 countries, and the shaded areas represent 95% confidence intervals.

International prices were largely stagnant in the last decades of the twentieth century, before rising steadily in the early 2000s and spiking sharply in a series of ‘food crises’ in 2007–2008 and 2010–2011, and more recently in 2021–2022 in the wake of the coronavirus disease 2019 (COVID-19) pandemic and the war in Ukraine (Fig. 1a). Consistent with rising international prices, the food component of the consumer price index (CPI) has risen, on average, 30% more than the total CPI in developing countries from January 2000 to September 2022 (Fig. 1b).

Yet whether these increases in real food prices translate into a true crisis of rising poverty in low- and middle-income countries (LMICs) is a matter of debate. Intuitively, higher food prices reduce disposable income for the poor because they spend large shares of their income on food (for example, 50% or more for the extremely poor), and even short-run income shocks can have severe long-run impacts on nutrition and health^1,2^. However, higher food prices can potentially also ‘increase’ incomes for households engaged in food production and marketing. As of 2013, 75% of the world’s poor (at the US$3.20-per-day poverty line) were rural^3^, and many of them would earn income from agriculture. Short-run simulation studies typically estimate changes in poverty based solely on whether a household is a net food consumer or a net food producer^4^, and almost invariably conclude that higher food prices increase poverty^5–13^. Such studies were highly influential among international agencies in the 2007–2008 crisis, and at least one recent simulation study on the 2021–2022 crisis draws similar conclusions to those earlier studies, with poverty rates rising by 27 million people (75% of them rural) in response to rising food, fuel and fertilizer prices in the 19 countries studied^14^.

However, the pessimistic conclusion that higher food prices increase poverty is questionable on a theoretical and a historical front. A previous study^15^ developed a theoretical and empirical model for rural India (a lower-middle-income country) illustrating how higher prices incentivize a food supply response from farmers, who raise their demand for labour, which puts upwards pressure on wages, to the benefit of the non-farm poor. This model also shows how this food supply and wage response reverses the pessimistic conclusions based on net food consumption measures alone. An economy-wide simulation model for Uganda (a low-income country) reaches similar conclusions^16^, while a series of retrospective World Bank national poverty assessments conducted several years after the 2007–2008 crisis concluded that higher food prices tended to reduce poverty in the studied countries, at least in rural areas^17–20^. A large cross-country panel-data analysis found that increases in domestic food prices predicted reductions in national poverty rates in developing countries over a 1–5 year time frame^21^. Accurate measurement of agricultural income and output is difficult in many LMICs, and methodological research in this area suggests that the standard 6–12 month recall period used to estimate agricultural output in farmer surveys results in a large underestimation of agricultural production^22,23^, leading to an overestimation of the extent to which rural households are net food consumers^24^.

Our study extends this literature in three important directions through a cross-country panel-data analysis of the relationships between changes in food prices, poverty and food production in 33 middle-income countries (MICs) over 2000–2019.

First, unlike previous econometric work and World Bank poverty assessments, our focus on annual data captures a reasonable definition of the ‘short run’, which has a span long enough to allow for the potential impacts of food supply and wage responses to materialize.

Second, the only previous panel analysis of changes in food prices and poverty modelled homogenous effects across countries^21^. However, while highly agrarian or rural populations may see national poverty rates decline as food prices increase, such a result is theoretically less likely in more urbanized or non-agrarian developing economies. Via interaction terms, our regression models allow the impacts of higher food prices to vary according to the extent of urbanization or non-agricultural employment.

Third, we provide an empirical exploration into a key mechanism by which higher food prices could reduce poverty, the stimulation of a short-run agricultural supply response. Crop farmers, especially, have flexibility to increase a wide range of inputs (seeds, fertilizer, labour and even planted area) in a short time span, if incentivized by higher prices. Theoretically, a strong short-run supply response is also a crucial catalyst for higher demand for unskilled labour and rising wage rates.

This study therefore provides a timely analysis of the nuanced linkages between food prices, agricultural production and poverty in a world where most of the poor are still rural and often heavily reliant on agriculture to earn a living.

## Results

### Descriptive results on poverty rates and real food prices

The mean annual change in the key outcome variable in our analysis, the US$3.20-per-day poverty headcount, over 2000–2019 was −0.43 percentage points across the 33 MICs. Our key explanatory variable is the annual change in the ratio of the food CPI to the non-food CPI, which, on average over our sample, increased by 0.83 percentage points per year, consistent with that of the larger sample of countries in Fig. 1b. Likewise, movements in this real domestic food price index vary over time in an expected fashion, with larger increases in years in which there were international price spikes (Fig. 1a). Supplementary Fig. 1 shows that in ‘food price crisis’ years, the real food price index increases by just over 5% on average, meaning food prices rose 5% faster than general prices of consumer goods and services. A simple bivariate regression suggests that international food price changes explain 23% of the total variation in the domestic food price index. Hence, international food price movement clearly explains a good deal of domestic food price variation, but there are clearly also idiosyncratic factors that influence the timing and extent of domestic food price movements, as we discuss below.

### Main regression results

Table 1 reports our main results for the association between changes in the US$3.20-per-day poverty headcount and changes in the food-to-non-food CPI ratio. Column 1 is a very basic linear first-differenced model, while column 2 adds year fixed effects. In both columns, the coefficient on changes in real food prices is negative, similar in magnitude and highly statistically significant, suggesting that increases in real domestic food prices predict reductions in poverty, on average. Regression 2 suggests that a 1 s.d. annual change in the food-to-non-food CPI ratio (approximately 5 percentage points) is associated with a modest 0.45-percentage-point reduction in the US$3.20-per-day poverty headcount.

**Table 1:** Associations between annual changes in poverty headcounts (US$3.20 per day) and percentage changes in real food prices in first-differenced regressions

	1	2	3	4	5	6
Food-to-non-food CPI ratio (% change)	−0.093***	−0.090***	−0.509***	−0.482***	−0.466***	−0.454***
	[−0.146, −0.040]	[−0.148, −0.032]	[−0.829, −0.188]	[−0.806, −0.159]	[−0.795, −0.137]	[−0.781, −0.128]
Food-to-non-food CPI ratio × urban share (%)			0.007***	0.007***		
			[0.002, 0.012]	[0.002, 0.012]		
Food-to-non-food CPI ratio × labour share in the non-agricultural sector (%)					0.005**	0.005**
					[0.001, 0.009]	[0.001, 0.009]
Year fixed effects?		Yes		Yes		Yes
*R*^2^	0.028	0.105	0.056	0.130	0.040	0.117
Number of observations	396	396	396	396	396	396
Number of countries	33	33	33	33	33	33

The outcome variable is the annual change in the poverty headcount at the US$3.20-per-day level, measured in percentage points. Ordinary least squares regression is based on equation (1) in columns 1 and 2 and based on equation (2) in columns 3 and 4. The unit of analysis is country–year. Values in square brackets are 95% confidence intervals and based on heteroskedasticity-robust standard errors. The urban- and labour-share variables are time invariant and measure the mean shares in a country over all available time periods.

***P < 0.01; **P < 0.05.

In columns 3 and 4, we estimate a model that introduces an interaction term between changes in real food prices and a country’s average urban population share. The estimated coefficient on changes in the food-to-non-food CPI ratio is now highly significant (*P* < 0.01) and still negative, whereas the interaction term is highly significant (*P* < 0.01) but positive, suggesting that the beneficial impacts of higher food prices on poverty reduction are attenuated or even reversed for countries with higher urban population shares.

**Fig. 2:**
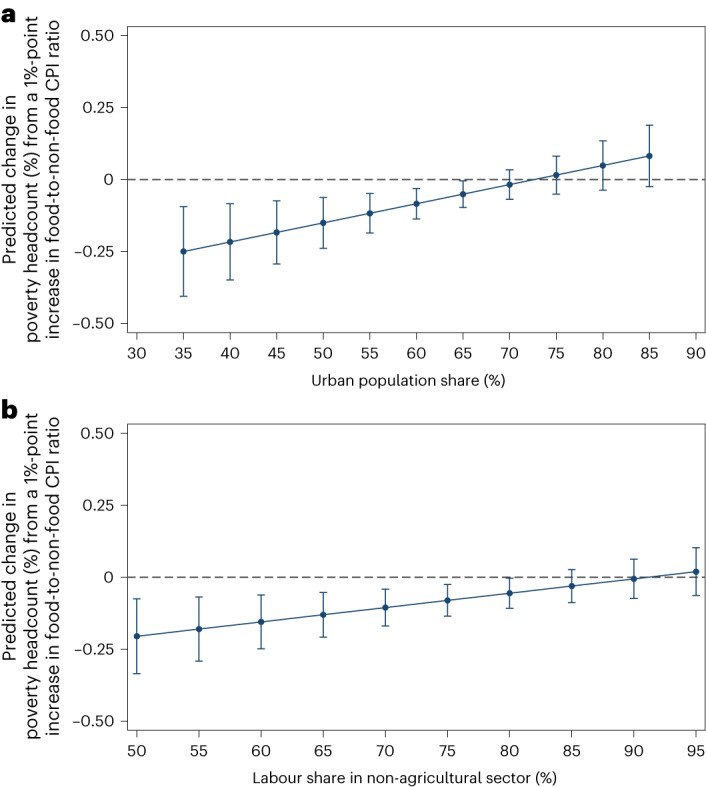
Predicted changes in the US$3.20-per-day poverty rate from a 1-percentage-point increase in the food-to-non-food CPI ratio, conditional on a country’s urban population share or non-agricultural labor share (with 95% confidence intervals). **a**, Predicted changes by urban population share. The regression line represents the predicted association of a 1%-point increase in the food-to-non-food CPI ratio with the $3.20-per-day poverty headcount conditional on the urban population share based on the coefficients reported in column 4 or 6 of Table 1. **b**, Predicted changes by labour share in the non-agricultural sector. The vertical capped lines represent 95% confidence intervals. The horizontal axis ranges correspond to minimum and maximum urban population and non-agricultural labour shares in our sample.

How should one interpret the magnitudes of these coefficients in the interaction models? The solid upward-sloping line in Fig. 2a represents the predicted change in poverty from a 1-percentage-point increase in real food prices conditional on the urban population share (across the range present in our data), based on the coefficients reported in column 4 of Table 1. The least urbanized MICs could expect economically and statistically significant reductions in poverty from large increases in real food prices. For example, a 5-percentage-point increase in food prices is associated with a 1.25-percentage-point reduction in poverty in the least urbanized countries in our dataset. At higher levels of urbanization (at around 70%), the benefits are no longer statistically different from zero.

**Fig. 3:**
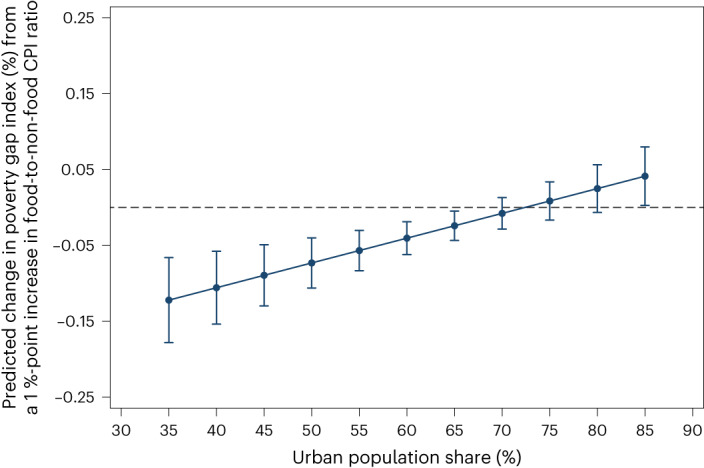
Predicted changes in the $3.20-per-day poverty gap index from a 1%-point increase in the food-to-non-food CPI ratio, with a first-difference estimator. The solid line shows the predicted association of a 1%-point increase in the food-to-non-food CPI ratio with the US$3.20-per-day poverty gap index (%) conditional on the urban population share based on the coefficients reported in column 4 of Supplementary Table 4. The vertical capped lines represent 95% confidence intervals.

The results are similar when we switch from urbanization as our ‘non-farm’ indicator to the share of the country’s labour force in non-agricultural employment. The regression results for the non-agricultural employment share interaction model (columns 5 and 6 in Table 1) correspond closely to the urbanization interaction effects reported in columns 3 and 4 of Table 1. Likewise, Fig. 2b shows that increases in real food prices are associated with reductions in poverty rates in countries that have relatively more people working in agriculture, but the relationship weakens in countries with fewer people working in agriculture. While the poverty headcount measures the share of the population falling into or out of poverty, the poverty gap tells us about changes in the depth of poverty. In Fig. 3, we observe that when we use the poverty gap measure as the dependent variable in our regression model, the key interaction coefficient between food price changes and urbanization still holds (Supplementary Table 4): at low levels of urbanization, a 5-percentage-point increase in the food-to-non-food CPI ratio is associated with a 0.6-percentage-point reduction in the poverty gap index, whereas at higher levels of urbanization, this association weakens and even becomes positive in highly urbanized MICs.

### Sensitivity tests

Next, we explore the robustness of the main regression results reported above.

First, when we include potential confounding factors, discussed above, the coefficients on the non-interacted and interacted terms remain stable and comparable to those reported in column 4 of Table 1 (Supplementary Table 5). Second, we re-estimate all our regression models using the US$1.90-per-day poverty headcount instead of the US$3.20-per-day poverty headcount (Supplementary Table 6 and Supplementary Fig. 2); the results remain similar to those in Table 1 and Fig. 2. Third, we apply a robust regression method to the first-difference estimator instead of the ordinary least square (OLS; Supplementary Table 7), which yields results qualitatively similar to the OLS results, although there is some modest attenuation of the coefficients after downweighing outliers. Similarly, the results are robust to a quantile regression approach^25^ that estimates the median of the outcome variable and is thus less sensitive to outliers than the OLS (Supplementary Table 8). Supplementary Table 9 also checks whether individual countries influence key associations, but they do not. Fourth, we make the right-hand-side variables in equation (2) interact with a binary variable equal to one if the survey was conducted during years when international food, fuel and fertilizer prices spiked (2007, 2008, 2010 and 2011), but these interactions are not statistically significant, indicating no special impacts during crisis years (Supplementary Table 10).

### Potential mechanisms

Why would increases in the real prices of retail foods be associated with reductions in poverty in more rural and more agrarian economies? Clearly, rural populations are poorer and more likely to be farmers and potential net food producers, but annual reductions in poverty presumably also require evidence that higher food prices stimulate an agricultural supply response, which in turn raises wage earnings. To test that hypothesis, we use a large panel (for the same 33 MICs) to model associations between growth rates of various measures of agricultural production and changes in the real domestic food price index.

**Fig. 4:**
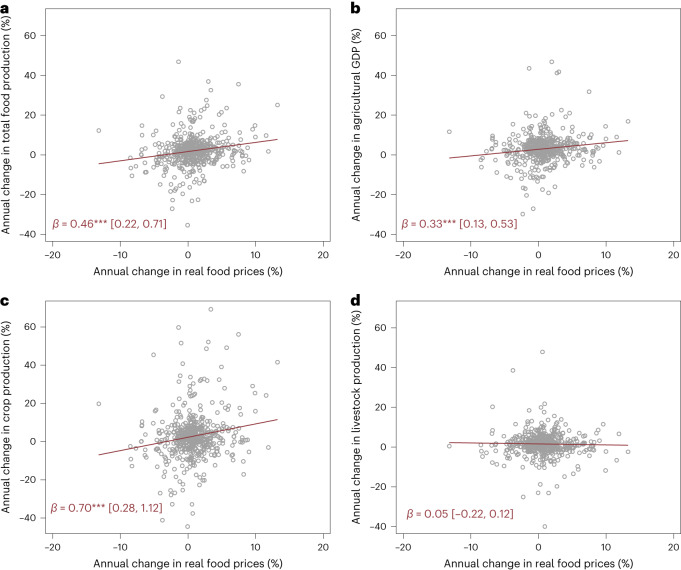
Scatterplots and linear regression fits of the associations between changes in real food prices (%) and changes in a country’s food production quantity index (a), agricultural GDP (b), crop production index (c) and livestock production index (d). **a**–**d**, Panel **a** uses the food production quantity index of the FAO as the dependent variable. Panel **b** uses agricultural GDP growth as the dependent variable. Panel **c** uses the crop production index as the dependent variable. Panel **d** uses the livestock production index of the FAO as the dependent variable. Each graph has a sample size of 501 observations from 33 MICs. Slope coefficients with 95% confidence intervals are reported in red, with statistical significance denoted with ***P < 0.01; **P < 0.05; *P < 0.1.

Figure 4a shows a scatterplot and linear regression fits of changes in the Food and Agriculture Organization (FAO) food production quantity index as a function of lagged changes in real retail food prices. The relationship is positive and statistically significant, suggesting that food production is, on average, highly responsive to retail food price changes in the short run. Figure 4b shows a positive but slightly weaker relationship for total agricultural gross domestic product (GDP) growth (that is, including non-food agricultural outputs), whereas Fig. 4c shows a strong positive association between crop production growth and real food price changes. Interestingly, but not surprisingly, livestock production is not correlated with domestic food price changes (Fig. 4d). Unlike crop production, where it is possible to expand a variety of inputs in the short run (for example, seeds, fertilizers, land, labour and machinery), expanding livestock production mostly requires acquiring larger herds or changing herd composition, which is almost impossible in the short run.

These bivariate results are robust to the inclusion of various controls (Supplementary Table 11) and to the robust regressor that downweighs the extreme values apparent in Fig. 4 (Supplementary Table 12), although those coefficients are smaller in magnitude than the OLS results. Specifically, a 5% increase in the real price of food predicts growth in total food production of around 1.95 percentage points in OLS regressions compared with 1.75 percentage points in robust regressions, and the corresponding responses for crop output growth are 3.3 percentage points and 1.8 percentage points.

These relatively strong short-run supply responses for crop production are likely to induce increased demand for unskilled labour and a relatively quick increase in wages^15^, although the speed and size of wage adjustments to rising food prices will be context specific and can also change over time with structural shifts in rural and urban labour markets (for example, urbanization and migration) and agricultural practices (for example, increased mechanization). One previous study found that rural wages in Bangladesh took only around 6 months to adjust to higher domestic food prices^24^, well within our annual time span, but another study from Bangladesh found that the association between food prices and farm wages has weakened over time^26^. Unfortunately, data on rural and urban wages for a wider array of countries are not available for more extensive testing of this mechanism, nor is there recent empirical evidence on other forms of rural non-farm spillovers from growth in domestic agricultural production.

## Discussion

While international food price spikes clearly have the potential to create problems for the urban poor, previous research has shown that higher domestic food prices tend to be poverty reducing at the national level, at least over the span of several years in cross-country panels^21^ or retrospective country case studies^17–20^. There is a missing middle in this evidence, however, because multi-year reductions in poverty say little about how long it takes for the incomes of the poor to improve or whether aggregate impacts differ according to the extent by which populations have transitioned out of rural areas or agricultural employment. Here we used a relatively short-run annual panel to robustly show that increases in food prices reduce poverty in less urbanized (more agrarian) middle-income economies and seem to have little or no impact on aggregate poverty in more urbanized economies.

We must resort to a combination of theory, previous findings and our own empirical evidence on the agricultural supply response to rising prices to help explain these findings. First, most of the world’s poor are still rural and engaged in livelihoods directly or indirectly connected to the agricultural economy; a World Bank study estimated that 75% of the US$3.20-per-day poor were living in rural areas in 2013 (ref. ^3^). Second, we showed that the food supply response to growth in domestic retail prices is quite strong, but is unsurprisingly driven solely by crop production, not livestock. Third, we know from previous research that this supply response involves an increased demand for labour, which at least raises rural wages, in the relatively short term.

While these findings collectively yield a compelling narrative of higher food prices stimulating rural poverty reduction, there are several limitations to our analysis.

First, we are compelled to use national-level poverty data and food price data to look at differential associations on rural and urban populations in a very indirect fashion. It is unfortunate that the World Bank does not yet report separate rural and urban poverty estimates for all countries to facilitate more granular research on this issue and many others of global importance, including targeting of anti-poverty interventions. Likewise, future research could separately analyse rural and urban price data, as rural and urban markets for food and non-food items could be poorly integrated in some settings.

Second, we find a robust conditional association between changes in poverty and changes in food prices, but do not establish causation. Domestic food price changes could be correlated with unobserved factors that independently influence poverty, including various shocks, but also government policies. Still, it is encouraging that more structural modelling approaches to this issue lead to broadly similar predictions on the differential impacts of higher food prices in rural and urban areas^15,16^.

Third, we focus on the welfare effects of food price increases, but not fertilizer or fuel price increases, which recent simulation analysis suggests could independently increase poverty^14^. That said, the 2007–2008 crisis also saw rapid increases in international food and fuel prices, but declining poverty at a global level and in various national poverty assessments^17–20^. More research is needed on this issue, including the complexities around the extent to which governments subsidize and stabilize fuel and fertilizer prices.

Fourth, we focus on an annual definition of the ‘short run’, which appears to encompass sufficient time for food supply and wage responses to higher food prices. More work is needed on high-frequency income, wage and food price data. Analyses of such data in Ethiopia^27^, and Kenya and Zambia^28^, show that rising prices did sharply reduce disposable income or urban populations in these countries, while the aforementioned analysis of data in rural and urban Bangladesh shows wage adjustment to higher prices in rural areas but not urban areas^24^. Still, while food price monitoring systems have been strengthened in the wake of the 2007–2008 crisis, international agencies and national governments have not extensively adopted high-frequency real-wage monitoring. They should do so^29^.

Fifth, our results may offer only limited insights into the outcomes of the 2021–2022 food crisis or welfare outcomes in any specific MIC. In contrast to 2007–2008, most LMICs in 2022 are in an especially weak fiscal position to deal with food, fuel and fertilizer inflation in the wake of the COVID-19 pandemic^30^. Indeed, it may be that the strong agricultural supply responses observed in LMICs in the wake of the 2007–2008 crisis will not easily be replicated because of the more limited fiscal capacity of LMIC governments to facilitate a strong supply response and because of exceptionally tight fertilizer supplies in 2022. In practice, welfare monitoring should be implemented at high frequency to gauge the welfare impacts of food inflation and other shocks^31^, all the more so because of climate change and the generally more volatile macroeconomic conditions prevailing in the global economy, and in light of the cost-effectiveness of phone-based welfare surveys during the COVID-19 pandemic^32^.

Bearing these limitations in mind, the current study builds on previous econometric and modelling research and illustrates the urbanization conditionality of the relationship between changes in poverty and changes in food prices in a broad swathe of MICs. Our key results are probably indicative of the fact that the bulk of the world’s poor—even in MICs—may still be predominantly rural and still frequently engaged in farming and that rural economies remain highly sensitive to positive or negative perturbations in the agricultural sector.

## Methods

We combined national data from various sources to form an annual panel dataset for 33 countries over 2000–2019 (and as such, did not require prior ethical approval) and conducted the analysis in Stata v17.

### Poverty, income and inequality measurement

We analysed 33 MICs with World Bank^33^ poverty measures reported on an annual basis (unfortunately, no low-income country has annual poverty estimates). However, as Supplementary Table 1 shows, these 33 MICs are characterized by large variation in average poverty headcounts at the US$3.20-per-day poverty line and the data are spread across Latin America (156 observations), Europe and Central Asia (193), and East Asia and the Pacific (40). Although some countries have more observations than others, we did ensure that each country’s time series does not contain gaps and does not switch between income-based poverty measures and consumption-based measures. We principally use the US$3.20-per-day poverty headcount as our dependent variable, but we also use the poverty gap, which measures the mean income of the poor as a percentage of the US$3.20-per-day poverty line. Supplementary Table 2 reports summary statistics showing that the average US$3.20-per-day poverty headcount in the dataset is 13%, but this varies between 0% for some observations and 75% as a maximum.

### Measurement of real food price changes

The direct effects of inflation on poverty are already addressed by the deflation of the income or expenditure measures used to calculate poverty. Here we instead study the potential effects of ‘real’ food price increases measured as annual changes in the ratio of the food CPI to the non-food CPI. This ratio can be calculated from a new International Monetary Fund (IMF)^34^ database containing disaggregated CPI indices and their associated CPI consumption basket weights, and indirectly estimated for an FAO^35^ CPI database that only reports food and total CPIs. For countries not reporting non-food CPIs (in either the IMF or the FAO database), we imputed weights from cross-country regressions of the IMF food CPI weights against the log of GDP per capita (on the basis that poorer populations have higher food expenditure shares), and then verified the predictive power of these imputations. The CPI estimates are reported on a monthly basis while the poverty data are annual. For any given poverty measurement year, we measure the real food price change between January of that year and January of the previous year to ensure that price changes always precede the poverty survey timings.

### Statistical analysis

We first used descriptive analysis to get a sense of the patterns in the data as well as trends in real food prices by estimating fitted regression lines of annual changes in food prices against binary variables capturing each year. We also produced a scatterplot of changes in the poverty headcount against changes in food prices, with linear regression lines for less urbanized and more urbanized countries (with the threshold at 60%, the average urban population share in our sample).

We then turned to more formal panel regression techniques by first modelling the poverty headcount or poverty gap index (pov_*i*,*t*_) in a country *i* in year *t* as a function of its real food price level (food price_*i*,*t*_) in the same year:

1 $${\Delta{\mathrm{pov}}}_{i,t}=\beta {\Delta{\mathrm{Food}}\,{\mathrm{price}}}_{i,t}+{{\mathrm{Year}}}_{t}+{\varepsilon }_{i,t}.$$

The estimated relationship between real food prices and poverty is given by *β*. The model purges time-invariant country characteristics using first differencing (that is, subtracting the previous year’s value from each observation). As a result, *β* is identified from annual within-country variation in real food price levels. Mindful of the limited degrees of freedom in our dataset, we explored sensitivity by including year fixed effects (Year_*t*_), that is, binary variables for each year in the dataset to control for time effects. These year fixed effects control for annual changes in the global macroeconomic environment that affect all countries in the dataset. The error term is captured in *ε*_*i,t*_.

We then added an interaction term between real food prices and a measure of the extent to which the population has transitioned out of rural areas or agricultural employment (‘non-farm’):

2 $$\begin{array}{l}\Delta {{\mathrm{pov}}}_{i,t}\\=\beta \Delta {{\mathrm{Food}}\,{\mathrm{price}}}_{i,t}+\gamma\left(\Delta {{\mathrm{Food}}\,{\mathrm{price}}}_{i,t}\times{{\mathrm{Non}}{\text-}{\mathrm{farm}}}_{i}\right)+{{\mathrm{Year}}}_{t}+{\varepsilon }_{i,t}.\end{array}$$

where *γ* refers to the coefficient on the interaction term. The rationale behind the ‘non-farm’ interaction is that households engaged in agriculture as farmers or farm workers could stand to benefit from higher food prices, while even non-farm rural populations could benefit from wage increases as demand for unskilled labour in rural areas increases. To measure ‘non-farm’, we used either the country’s urban population share or its non-agricultural employment share. It is not obvious, a priori, whether urbanization or non-agricultural employment shares are the best way to capture heterogenous food price–poverty associations across countries; both are conceptually relevant, so exploring sensitivity to this choice is important. Also, note that these two ‘non-farm’ non-indicators are averages, as these indicators change little over time and many values are imputed between infrequent censuses or labour force surveys. Using a within-country average for ‘non-farm’ means that any cross-country variation is removed by first differencing, so ‘non-farm’ does not enter equation (2) as a separate variable, only as an interaction term. Another point to note is that these two ‘non-farm’ indicators are highly correlated with each other, with a correlation coefficient of 0.76. Urban population shares vary markedly between 36% and 84% across the sampled countries, as do non-agricultural labour shares (51% to 95%).

Ideally, we would exploit exogenous variation in real food prices to identify a causal impact, but in reality, food prices can be affected by government policies and domestic shocks (for example, droughts, conflict, macroeconomic crises) that could affect poverty through non-price mechanisms (for example, droughts could affect food prices and independently reduce farm incomes), leading to omitted-variable bias. To explore the potential problem of confounding factors, we appended equation (2) as follows:

3 $$\begin{array}{l}{\Delta{\mathrm{pov}}}_{i,t}\\=\beta \Delta {{\mathrm{Food}}\,{\mathrm{price}}}_{i,t}+\gamma \left(\Delta {{\mathrm{Food}}\,{\mathrm{price}}}_{i,t}\times{{\mathrm{Non}}{\text-}{\mathrm{farm}}}_{i}\right)+{\Delta \textbf{X}}_{i,t}^{{\prime} }\delta +{{\mathrm{Year}}}_{t}+{\varepsilon }_{i,t},\end{array}$$

where **X**′_*i,t*_ represents a vector of time-varying control variables, including changes in non-agricultural GDP, money supply, exchange rates, terms of trade, the number of battle-related deaths and surface temperature change relative to 1951–1980 in country *i* in year *t*, and *δ* represents the coefficients on these variables. The temperature variable was sourced from the FAO^36^ while all the other control variables were obtained from the World Bank^37^. The selection of the control variables was motivated by an earlier cross-country panel analysis of food prices and poverty over the longer term^21^, with the addition of temperature changes as a newly available indicator. To explore the sensitivity of our estimates to these control variables, we added them into the model one at a time as well as together. Supplementary Table 2 provides summary statistics for these control variables.

To explore the mechanisms through which real food price inflation predicts poverty, we tested whether the countries in our sample experienced changes in agricultural output in response to lagged increases in the real retail price of food. To do so, we regressed annual percentage change in agricultural output ($\Delta$ ag_output_*i*,*t*_) as a function of annual changes in real food prices:

4 $${\Delta {\rm{ag}}\_{\mathrm{output}}}_{i,t}=\beta \Delta {{\mathrm{Food}}\,{\mathrm{price}}}_{i,t}+{\Delta \textbf{X}}_{i,t}^{{\prime} }\delta +{{\mathrm{Year}}}_{t}+{\varepsilon }_{i,t}.$$

As measures of agricultural output, we used agricultural GDP (measured in 2015 constant USD), food production, crop production and livestock production. The food, crop and livestock production indices were sourced from the FAO^36^, and they are weighted sums of quantities produced in the country, where commodity-specific weights are based on average international commodity prices in 2014–2016. The control variables are the same as those used in equation (2). We restricted the analysis to the same 33 MICs, but as we are not constrained by the availability of poverty data, we can considerably extend the country-specific time series to estimate equation (4). After excluding two extreme outliers that appear to be measurement error, we have a sample of 501 observations (Supplementary Table 3).

Our main regressions were estimated using OLS. On the basis of post-regression diagnostic tests, the null of homoskedasticity is rejected whereas the null of no autocorrelation in the residuals is not. Therefore, we used non-clustered standard errors that are robust to heteroskedasticity^38^. Because there is evidently some noise in the World Bank poverty estimates and other indicators used in the analyses, we also explored the sensitivity of our estimates to using a robust regressor to downweigh influential outliers. All statistical analyses were implemented in Stata v17.

### Reporting summary

Further information on research design is available in the Nature Portfolio Reporting Summary linked to this article.

## Supplementary information

Supplementary Information Supplementary Figs. 1 and 2 and Tables 1–12.

Reporting Summary

## Data Availability

All the data used in this analysis are publicly available, and the specific data for replicating our analysis are available online^39^.

## Code availability

The Stata v17 code for replicating our analysis is available online^39^.
