# Supplementary material for: Higher food prices can reduce poverty and stimulate growth in food production
Source: Nat Food. 2023 Aug 10;4(8):699–706. doi: 10.1038/s43016-023-00816-8 (PMC10444620; doi:10.1038/s43016-023-00816-8)
Supplement: Supplementary file 1 — Supplementary Figs. 1 and 2 and Tables 1–12. [file 43016_2023_816_MOESM1_ESM.pdf]

nature food

Article

https://doi.org/10.1038/s43016-023-00816-8

Higher food prices can reduce poverty and stimulate growth in food production

In the format provided by the authors and unedited

Supplement

Contents

Supplementary Table 1. Composition of the dataset 2

Supplementary Table 2. Summary statistics for key indicators in the poverty analysis: 396 years in 33 countries 3

Supplementary Table 3. Summary statistics for the agricultural supply response regression analysis for the same 31 middle income country sample covering 2000-2019 4

Supplementary Figure 1. Predicted annual changes in food/nonfood CPI ratios over 2002-2019 in countries in the final dataset, with 95% confidence intervals 5

Supplementary Table 4. Associations between changes in the $3.20/day poverty gap index (%) and percentage changes in real food prices in first-differenced regressions 6

Supplementary Table 5. Associations between changes in poverty headcounts ($3.20/day) and percentage changes in real food prices in first-differenced regressions with time-varying controls 7

Supplementary Table 6. Associations between changes in the $1.90/day headcounts and percentage changes in real food prices in first-differenced regressions 8

Supplementary Figure 2. Predicted associations of a 1%–point increase in the food/nonfood CPI ratio on the change in the $1.90/day poverty rate from first differenced regressions 9

Supplementary Table 7. Associations between changes in poverty headcounts ($3.20/day) and percentage changes in real food prices in first-differenced models estimated with the robust regressor to downweigh outliers 10

Supplementary Table 8. Associations between changes in poverty headcounts ($3.20/day) and percentage changes in real food prices in first-differenced models estimated with the median regression that is less sensitive to outlier than the OLS 11

Supplementary Table 9. Re-estimating column 3 in Table 1 by omitting one country at a time from the dataset 12

Supplementary Table 10. Interactions between domestic food price changes and 'global food crises years' 13

Supplementary Table 11. Least squares first-differenced regressions of changes in various agricultural output measures against growth in real food prices and various controls 14

Supplementary Table 12. Robust first-differenced regressions of changes in various agricultural output measures against growth in real food prices and various controls 15

Supplementary Table 1. Composition of the dataset

Country	N	Years	Mean poverty rate, $3.20/day	Mean GDP per capita	World Bank income status
Albania	3	2015–2017	8.6	4,098	Upper middle
Armenia	18	2002–2019	16.2	3,093	Upper middle
Azerbaijan	3	2002–2004	0.5	1,917	Upper middle
Belarus	18	2002–2019	1.8	5,162	Upper middle
Bolivia	8	2012–2019	11.9	3,054	Lower middle
Brazil	8	2012–2019	8.5	8,813	Upper middle
Bulgaria	10	2009–2018	4.3	6,938	Upper middle
China	6	2011–2016	13.0	7,311	Upper middle
Colombia	11	2009–2019	14.3	5,860	Upper middle
Costa Rica	19	2001–2019	5.8	10,271	Upper middle
Dominican Republic	19	2001–2019	11.0	5,863	Upper middle
Ecuador	16	2004–2019	14.8	5,576	Upper middle
El Salvador	18	2001–2019	16.6	3,446	Lower middle
Georgia	19	2001–2019	25.6	3,194	Upper middle
Honduras	18	2002–2019	33.9	2,154	Lower middle
Indonesia	19	2001–2019	45.3	2,783	Upper middle
Iran, Islamic Rep	5	2014–2018	3.1	5,249	Upper middle
Kazakhstan	17	2002–2018	5.7	8,857	Upper middle
Kosovo	8	2010–2017	6.8	3,307	Upper middle
Kyrgyz Republic	18	2001–2019	28.0	989	Lower middle
Mexico	2	2005–2006	14.5	9,010	Upper middle
Moldova	5	2014–2018	1.1	2,934	Lower middle
Mongolia	2	2011–2012	5.3	3,182	Lower middle
Montenegro	9	2006–2014	1.1	5,990	Upper middle
North Macedonia	8	2011–2018	11.0	4,768	Upper middle
Paraguay	18	2002–2019	11.7	4,762	Upper middle
Peru	19	2001–2019	18.2	5,090	Upper middle
Russian Federation	18	2001–2018	2.4	8,360	Upper middle
Serbia	8	2003–2010	2.0	4,702	Upper middle
Thailand	13	2007–2019	1.5	5,613	Upper middle
Turkey	17	2003–2019	4.9	9,457	Upper middle
Ukraine	14	2006–2019	0.4	2,359	Lower middle
West Bank and Gaza	2	2010–2011	3.1	3,028	Lower middle
**Total**	**396**				

Source: See the main text for sources.

Supplementary Table 2. Summary statistics for key indicators in the poverty analysis: 396 years in 33 countries

Variable	N	Mean	Std. dev.	Min	Max
Key outcome indicators					
Poverty headcount (%), $3.20/day	396	13.0	13.7	0.0	74.6
Poverty gap index (%), $3.20/day	396	4.3	5.1	0.0	26.6
Key explanatory variables					
Food/nonfood CPI ratio (%)	396	95.3	11.8	57.3	134.7
Mean urban population share (%)	396	63.2	11.7	35.3	86.8
Mean labor share in non-agricultural sector (%)	396	75.3	11.6	51.2	95.2
Control variables					
Non-agricultural GDP per capita (2015 US$)	386	4,830.4	2,580.8	487	12,200
Broad money (% GDP)	394	48.3	27.6	11.14	207.67
Exchange rate index (LCU/US$) (2011=100)	394	121.6	55.0	36.00	420.49
Battle-related deaths per 10,000 people	396	1.1	2.1	0.00	8.38
Net barter terms of trade index (2000=100)	379	118.0	30.5	67.35	223.51
Temperature change relative to 1951–1980	388	1.10	0.57	-0.26	2.81

Notes: Broad money is the sum of currency outside banks**.** Std. Dev. = Standard Deviation. LCU = Local currency unit. See main text for sources.

Supplementary Table 3. Summary statistics for the agricultural supply response regression analysis for the same 31 middle income country sample covering 2000-2019

Variable	N	Mean	SD	Min	Max
Annual change in agricultural GDP (%)[a]	501	3.09	7.23	-29.78	46.81
Annual change in food production (%)[b]	501	2.01	8.00	-35.36	46.84
Annual change in crop production (%)[b]	501	2.82	12.54	-44.45	69.26
Annual change in livestock production (%)[b]	501	1.55	6.17	-39.98	47.85
Annual change in real food price (%)[c]	501	0.77	3.15	-13.18	13.24
Annual change in non-agricultural GDP (%)[a]	501	4.33	3.99	-14.55	28.4
Temperature change relative to 1951-1980 average (C) [a]	501	0.61	0.60	0.0	2.81
Annual change in log of battle deaths per 1000 people[a]	501	0.15	0.89	0.0	14.96
Annual change in broad money supply (%)[a]	501	16.92	15.91	-20.01	154.39
Annual change in terms of trade (%)[a]	501	0.01	0.09	-0.37	.42
Annual change in exchange rate (%)[a]	501	1.12	24.42	-1.0	546.53

Source: a. World Bank[26]; b. FAO[37]; c. IMF[36].

Supplementary Figure 1. Predicted annual changes in food/nonfood CPI ratios over 2002-2019 in countries in the final dataset, with 95% confidence intervals

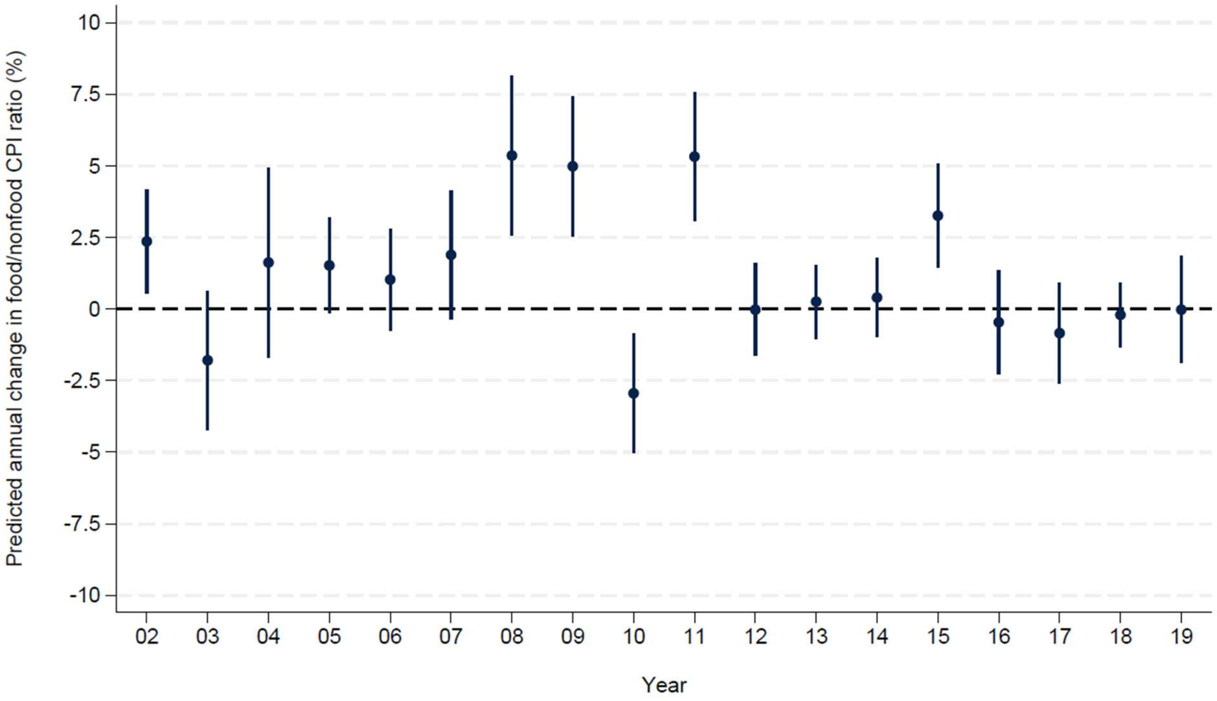

Notes: The dots represent average deviation in food/nonfood CPI ratio and the lines are 95%-confidence intervals. These results are derived from an ordinary least squares (OLS) regression of the annual change in the food/nonfood CPI ratio against annual binary variables, with confidence intervals based on heteroskedasticity robust standard errors. N = 396 observations from 33 countries.

Supplementary Table 4. Associations between changes in the $3.20/day poverty gap index (%) and percentage changes in real food prices in first-differenced regressions

	(1)	(2)	(3)	(4)
Food/nonfood CPI ratio (%)	-0.041***<br>[-0.063,-0.019]	-0.043***<br>[-0.068,-0.019]	-0.249***<br>[-0.362,-0.136]	-0.236***<br>[-0.351,-0.122]
Food/nonfood CPI ratio (%) * Urban share			0.004***<br>[0.002,0.005]	0.003***<br>[0.002,0.005]
Year fixed effects?		Yes		Yes
R^2	0.029	0.109	0.068	0.141
Number of observations	396	396	396	396
Number of countries	33	33	33	33

Notes: Outcome variable is poverty gap index at $3.20/day level, measured in %. OLS regression based on equation (1) in columns 1–2 and based on equation (2) in columns 3–4. Unit of analysis is country-year. Heteroskedasticity robust standard errors reported in parentheses. Statistical significance based on two-sided t-test and denoted with *** p<0.01, ** p<0.05, * p<0.1. Urban share variable is time-invariant and measures the mean share of urban population relative to total population over time.

Supplementary Table 5. Associations between changes in poverty headcounts ($3.20/day) and percentage changes in real food prices in first-differenced regressions with time-varying controls

	(1)	(2)	(3)	(4)	(5)	(6)	(7)
Food/nonfood CPI ratio (%)	-0.423***	-0.487***	-0.483***	-0.490***	-0.469***	-0.484***	-0.435**
	[-0.744,-0.103]	[-0.817,-0.158]	[-0.807,-0.158]	[-0.817,-0.163]	[-0.792,-0.146]	[-0.809,-0.160]	[-0.769,-0.102]
Food/nonfood CPI ratio* Urban share	0.006**	0.007***	0.007***	0.007***	0.006***	0.007***	0.006**
	[0.001,0.011]	[0.002,0.012]	[0.002,0.012]	[0.002,0.012]	[0.002,0.011]	[0.002,0.012]	[0.001,0.011]
(log) Non-agricultural GDP per capita	-13.582***						-13.722**
	[-23.627,-3.537]						[-24.875,-2.569]
Broad money (% GDP)		-0.026					-0.049
		[-0.104,0.052]					[-0.145,0.047]
Exchange rate index (LCU/US$)			0.000				-0.001
			[-0.006,0.007]				[-0.006,0.003]
(log) Battle-related deaths				-0.173			-0.186*
				[-0.398,0.053]			[-0.404,0.032]
Net barter terms of trade index					-0.006		-0.007
					[-0.032,0.019]		[-0.034,0.020]
Temperature change relative to 1951–1980						0.051	0.088
						[-0.373,0.475]	[-0.392,0.569]
Year fixed effects?		Yes	Yes	Yes	Yes	Yes	Yes
R^2	0.159	0.132	0.129	0.134	0.131	0.129	0.169
Number of observations	385	393	394	396	379	388	355
Number of countries	33	33	32	33	31	30	30

Notes: Outcome variable is poverty headcount at $3.20/day level, measured in %. OLS regression based on equation (3). Unit of analysis is country-year. 95%-confidence intervals based on heteroskedasticity robust standard errors reported in brackets. Statistical significance based on two-sided t-test and denoted with *** p<0.01, ** p<0.05, * p<0.1. Urban share variable is time-invariant and measures the mean share of urban population relative to total population over time.

Supplementary Table 6. Associations between changes in the $1.90/day headcounts and percentage changes in real food prices in first-differenced regressions

	(1)	(2)	(3)	(4)
Food/nonfood CPI ratio (%)	-0.051***	-0.057***	-0.327***	-0.310***
	[-0.081,-0.022]	[-0.089,-0.025]	[-0.477,-0.177]	[-0.461,-0.160]
Food/nonfood CPI ratio (%) * Urban share			0.005***	0.004***
			[0.002,0.007]	[0.002,0.007]
Year fixed effects?		Yes		Yes
R^2	0.027	0.110	0.066	0.142
Number of observations	396	396	396	396
Number of countries	33	33	33	33

Notes: Outcome variable is poverty headcount at $1.90/day level, measured in %. OLS regression based on equation (1) in columns 1–2 and based on equation (2) in columns 3–4. Unit of analysis is country-year. Heteroskedasticity robust standard errors reported in parentheses. Statistical significance based on two-sided t-test and denoted with *** p<0.01, ** p<0.05, * p<0.1. Urban share variable is time-invariant and measures the mean share of urban population relative to total population over time.

Supplementary Figure 2. Predicted associations of a 1%–point increase in the food/nonfood CPI ratio on the change in the $1.90/day poverty rate from first differenced regressions

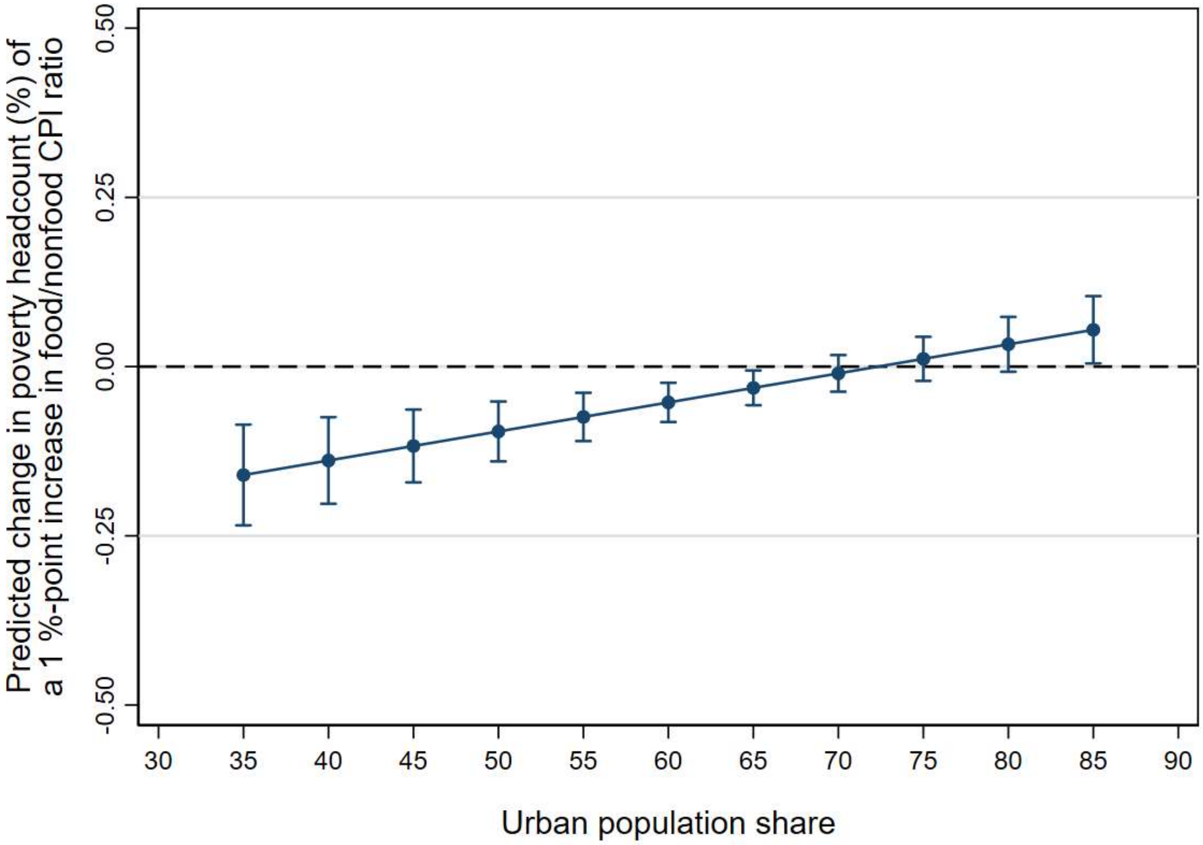

Notes: The dots represent the predicted association of a 1%–point increase in the food/nonfood CPI ratio on $1.90 poverty headcount conditional on the urban population share based on the OLS coefficients reported in Column 4 of Supplementary Table 4. The vertical capped lines represent 95%–confidence intervals based on heteroskedasticity robust standard errors. N = 396 observations from 33 countries.

Supplementary Table 7. Associations between changes in poverty headcounts ($3.20/day) and percentage changes in real food prices in first-differenced models estimated with the robust regressor to downweigh outliers

	(1)	(2)	(3)	(4)
Food/nonfood CPI ratio (%)	-0.072***	-0.067***	-0.396***	-0.363***
	[-0.105,-0.039]	[-0.103,-0.031]	[-0.538,-0.255]	[-0.507,-0.220]
Food/nonfood CPI ratio (%) * Urban share			0.005***	0.005***
			[0.003,0.008]	[0.002,0.007]
Year fixed effects?		Yes		Yes
R^2	0.044	0.161	0.110	0.213
Number of observations	396	396	396	396
Number of countries	33	33	33	33

Notes: Outcome variable is poverty headcount at $3.20/day level, measured in %. Robust regression based on equation (1) in columns 1–2 and based on equation (2) in columns 3–4. Unit of analysis is country-year. Robust regression standard errors reported in parentheses. Statistical significance based on two-sided t-test and denoted with *** p<0.01, ** p<0.05, * p<0.1. Urban share variable is time-invariant and measures the mean share of urban population relative to total population over time. Regression is implemented using the *rreg* command in Stata v17™.

Supplementary Table 8. Associations between changes in poverty headcounts ($3.20/day) and percentage changes in real food prices in first-differenced models estimated with the median regression that is less sensitive to outlier than the OLS

	(1)	(2)	(3)	(4)
Food/nonfood CPI ratio (% change)	-0.085***	-0.074***	-0.494***	-0.424***
	[-0.126, -0.044]	[-0.119, -0.030]	[-0.764, -0.224]	[-0.683, -0.166]
Food/nonfood CPI ratio * Urban share (%)			0.007***	0.006***
			[0.003, 0.011]	[0.002, 0.010]
Year fixed effects?	No	Yes	No	Yes
Observations	396	396	396	396

Notes: Median regression based on quantile regression approach. Outcome variable is the annual change in the poverty headcount at $3.20/day level, measured in percentage points. OLS regression based on equation (1) in columns 1–2 and based on equation (2) in columns 3–4. Unit of analysis is country–year. 95–% confidence intervals are reported in brackets and based on bootstrapping with 1,000 repetitions. Statistical significance based on two-sided t-test and denoted with *** p<0.01, ** p<0.05, * p<0.1. The urban share variable is time–invariant and measure the mean shares over time.

Supplementary Table 9. Re-estimating column 3 in Table 1 by omitting one country at a time from the dataset

Country omitted:	Coefficient, Food/nonfood CPI ratio (%)	Coefficient, Food/nonfood CPI ratio (%) * Urban share	N	R^2
Albania	-0.510***	0.007***	393	0.053
Armenia	-0.509***	0.007***	378	0.062
Azerbaijan	-0.502***	0.007***	393	0.059
Bulgaria	-0.513***	0.007***	386	0.056
Belarus	-0.522***	0.007***	378	0.057
Bolivia	-0.509***	0.007***	388	0.056
Brazil	-0.512***	0.007***	388	0.056
China	-0.491***	0.007***	390	0.053
Colombia	-0.515***	0.007***	385	0.056
Costa Rica	-0.495***	0.007***	377	0.056
Dominican Republic	-0.492***	0.007***	377	0.058
Ecuador	-0.509***	0.007***	380	0.057
Georgia	-0.520***	0.007***	377	0.064
Honduras	-0.480***	0.007***	378	0.049
Indonesia	-0.488***	0.007***	377	0.055
Iran	-0.499***	0.007***	391	0.056
Kazakhstan	-0.508***	0.007***	379	0.063
Kyrgyz Republic	-0.568***	0.008***	378	0.033
Moldova	-0.514***	0.007***	391	0.057
Mexico	-0.510***	0.007***	394	0.056
North Macedonia	-0.509***	0.007***	388	0.056
Montenegro	-0.509***	0.007***	387	0.056
Mongolia	-0.510***	0.007***	394	0.056
Peru	-0.517***	0.007***	377	0.058
Paraguay	-0.513***	0.007***	378	0.061
West Bank and Gaza	-0.510***	0.007***	394	0.056
Russian Federation	-0.504***	0.007***	378	0.056
El Salvador	-0.508***	0.007***	378	0.058
Serbia	-0.514***	0.007***	388	0.055
Thailand	-0.554***	0.008***	383	0.062
Turkey	-0.512***	0.007***	379	0.056
Ukraine	-0.510***	0.007***	382	0.055
Kosovo	-0.496***	0.007***	388	0.053

Notes: Outcome variable is poverty headcount at $3.20/day level, measured in %. OLS regression based on equation (2). Unit of analysis is country-year. Heteroskedasticity robust standard errors reported in parentheses. Statistical significance based on two-sided t-test and denoted with *** p<0.01, ** p<0.05, * p<0.1.

Supplementary Table 10. Interactions between domestic food price changes and ‘global food crises years’

	(1)	(2)	(3)
Food/nonfood CPI ratio (%)	-0.090***	-0.088**	-0.501***
	[-0.148,-0.032]	[-0.157,-0.019]	[-0.842,-0.159]
Food/nonfood CPI ratio (%) * ‘Global crisis year’		-0.007	0.009
		[-0.134,0.119]	[-0.107,0.125]
Food/nonfood CPI ratio (%) * Urban share			0.007***
			[0.002,0.012]
Food/nonfood CPI ratio (%) * ‘Global crisis year’ * Urban share			0.000
			[-0.000,0.000]
Year Fixed effects?	Yes	Yes	Yes
R^2	0.105	0.105	0.131
Observations	396	396	396
Number of countries	33	33	33

Notes: Outcome variable is poverty headcount at $3.20/day level, measured in %. OLS regression based on equation (1) in columns 1–2 and based on equation (2) in columns 3–4, appended with interactions difference terms capturing global food crises years (2007, 2008, 2010 and 2011). The un-interacted ‘crisis year’ variable is absorbed in the year fixed effects. Unit of analysis is country-year. Heteroskedasticity robust standard errors reported in parentheses. Statistical significance based on two-sided t-test and denoted with *** p<0.01, ** p<0.05, * p<0.1. Urban share variable is time-invariant and measures the mean share of urban population relative to total population over time.

Supplementary Table 11. Least squares first-differenced regressions of changes in various agricultural output measures against growth in real food prices and various controls

	(1) Agricultural GDP	(2) Food production	(3) Crop production	(4) Livestock production
Food/nonfood CPI ratio [%]	0.31**	0.39***	0.65***	-0.10
	[0.06 - 0.55]	[0.10 - 0.69]	[0.18 - 1.11]	[-0.32 - 0.12]
Non-agricultural GDP [%]	0.09	0.11	0.12	0.18***
	[-0.09 - 0.28]	[-0.08 - 0.29]	[-0.19 - 0.43]	[0.05 - 0.31]
Temperature change [C]	-0.41	0.23	0.75	-0.07
	[-1.99 - 1.16]	[-1.37 - 1.84]	[-1.54 - 3.05]	[-1.25 - 1.12]
Log of battle deaths	-0.45**	-0.84**	-1.16**	-0.39**
	[-0.85 - -0.06]	[-1.50 - -0.18]	[-2.09 - -0.22]	[-0.71 - -0.07]
Money supply [%]	-0.00	-0.05**	-0.03	-0.05**
	[-0.04 - 0.03]	[-0.09 - -0.00]	[-0.10 - 0.04]	[-0.09 - -0.01]
Terms of trade [%]	-4.90	-5.74	-7.10	0.26
	[-12.05 - 2.25]	[-13.28 - 1.80]	[-22.46 - 8.26]	[-6.22 - 6.74]
Exchange rate [%]	-0.01**	-0.01	-0.03***	0.02***
	[-0.02 - -0.00]	[-0.02 - 0.00]	[-0.05 - -0.02]	[0.01 - 0.03]
Year fixed effects?	Yes	Yes	Yes	Yes
Observations	501	501	501	501
R-squared	0.10	0.11	0.11	0.06

Notes: OLS regression based on equation [4]. Unit of analysis is country-year. 95%-confidence intervals based on heteroskedasticity robust standard errors reported in brackets. Statistical significance based on two-sided t-test and denoted with *** p<0.01, ** p<0.05, * p<0.1.

Supplementary Table 12. Robust first-differenced regressions of changes in various agricultural output measures against growth in real food prices and various controls

	(1) Agricultural GDP	(2) Food production	(3) Crop production	(4) Livestock production
Food/nonfood CPI ratio [%]	0.21***	0.35***	0.36***	0.04
	[0.06 - 0.36]	[0.18 - 0.52]	[0.10 - 0.62]	[-0.09 - 0.17]
Non-agricultural GDP [%]	0.16***	0.32***	0.36***	0.22***
	[0.04 - 0.29]	[0.17 - 0.46]	[0.14 - 0.58]	[0.10 - 0.33]
Temperature change [C]	0.21	0.45	0.39	-0.13
	[-0.67 - 1.08]	[-0.54 - 1.44]	[-1.11 - 1.88]	[-0.88 - 0.63]
Log of battle deaths	-0.34	-0.59**	-0.75*	-0.40*
	[-0.80 - 0.13]	[-1.11 - -0.06]	[-1.54 - 0.04]	[-0.80 - 0.00]
Money supply [%]	-0.01	-0.05***	-0.06**	-0.03**
	[-0.04 - 0.02]	[-0.08 - -0.01]	[-0.11 - -0.01]	[-0.06 - -0.01]
Terms of trade [%]	-2.81	-6.95**	-9.44**	1.06
	[-7.88 - 2.26]	[-12.69 - -1.21]	[-18.11 - -0.77]	[-3.31 - 5.44]
Exchange rate [%]	-0.01	1.71	1.83	1.37
	[-0.03 - 0.01]	[-1.78 - 5.20]	[-3.44 - 7.09]	[-1.29 - 4.03]
Year fixed effects?	Yes	Yes	Yes	yes
R^2	0.10	0.14	0.10	0.11
Number of observations	502	502	502	502
Number of countries	33	33	33	33

Notes: Robust regression based on equation [4]. Unit of analysis is country-year. 95%-confidence intervals based on robust regression standard errors reported in brackets. Statistical significance based on two-sided t-test and denoted with *** p<0.01, ** p<0.05, * p<0.1. Regression is implemented using the *rreg* command in Stata v17™.
