# Supplementary material for: Higher food prices can reduce poverty and stimulate growth in food production
Source: Nat Food. 2023 Aug 10;4(8):699–706. doi: 10.1038/s43016-023-00816-8 (PMC10444620; doi:10.1038/s43016-023-00816-8)
Supplement: Supplementary file 2 — Reporting Summary [file 43016_2023_816_MOESM2_ESM.pdf]

Corresponding author(s): Derek Headey

Last updated by author(s): Jul 6, 2023

Reporting Summary

Nature Research wishes to improve the reproducibility of the work that we publish. This form provides structure for consistency and transparency in reporting. For further information on Nature Research policies, see our Editorial Policies and the Editorial Policy Checklist.

Statistics

For all statistical analyses, confirm that the following items are present in the figure legend, table legend, main text, or Methods section.

n/a	Confirmed	
☐	☒	The exact sample size (n) for each experimental group/condition, given as a discrete number and unit of measurement
☒	☐	A statement on whether measurements were taken from distinct samples or whether the same sample was measured repeatedly
☐	☒	The statistical test(s) used AND whether they are one- or two-sided<br>*Only common tests should be described solely by name; describe more complex techniques in the Methods section.*
☐	☒	A description of all covariates tested
☒	☐	A description of any assumptions or corrections, such as tests of normality and adjustment for multiple comparisons
☐	☒	A full description of the statistical parameters including central tendency (e.g. means) or other basic estimates (e.g. regression coefficient) AND variation (e.g. standard deviation) or associated estimates of uncertainty (e.g. confidence intervals)
☐	☒	For null hypothesis testing, the test statistic (e.g. F, t, r) with confidence intervals, effect sizes, degrees of freedom and P value noted<br>*Give P values as exact values whenever suitable.*
☒	☐	For Bayesian analysis, information on the choice of priors and Markov chain Monte Carlo settings
☒	☐	For hierarchical and complex designs, identification of the appropriate level for tests and full reporting of outcomes
☒	☐	Estimates of effect sizes (e.g. Cohen's d, Pearson's r), indicating how they were calculated

Our web collection on statistics for biologists contains articles on many of the points above.

Software and code

Policy information about availability of computer code

Data collection	Stata Version 17 was used to merge together IMF and FAO food price data with World Bank indicators at the national level, to form a multi-country panel. All data used were publicly available. A full replication code is provided at https://zenodo.org/record/8119555
Data analysis	Stata Version 17 was used to calculate descriptive statistics and implemented non-parametric and parametric regression analysis.

For manuscripts utilizing custom algorithms or software that are central to the research but not yet described in published literature, software must be made available to editors and reviewers. We strongly encourage code deposition in a community repository (e.g. GitHub). See the Nature Research guidelines for submitting code & software for further information.

Data

Policy information about availability of data

All manuscripts must include a data availability statement. This statement should provide the following information, where applicable:

- Accession codes, unique identifiers, or web links for publicly available datasets
- A list of figures that have associated raw data
- A description of any restrictions on data availability

This analysis uses multiple sources of national level representative data which were merged into a single dataset, which is available here along with our Stata v 17 replication codes: https://zenodo.org/record/8119555
This study constructed cross-country panel datasets from 7 global datasets, all of which are publicly available and listed below.
1 IMF. International Commodity Price Database, The International Monetary Fund, https://www.imf.org/en/Research/commodity-prices> (2022).
2 FAO. FAO Food Price Index Database, Food and Agriculture Organisation, https://fenix.fao.org/faostat/internal/en/#data/CP> (2022).
3 IMF. IMF Consumer Price Index Database, https://data.imf.org/?sk=4FFB52B2-3653-409A-B471-D47B46D904B5&sId=1485878855236> (2022).
4 FAO. FAOSTAT Consumer Price Indices, Food and Agriculture Organization, https://www.fao.org/faostat/en/> (2022).

5 World Bank. Poverty and Inequality Platform, The World Bank, https://pip.worldbank.org/home> (2022).
6 World Bank. World Development Indicators Online, The World Bank, http://devdata.worldbank.org/dataonline/> (2022).
7 FAO. FAOSTAT Production Indices, Food and Agriculture Organization, https://fenix.fao.org/faostat/internal/en/#data/QI (2022)

Field-specific reporting

Please select the one below that is the best fit for your research. If you are not sure, read the appropriate sections before making your selection.

[ ] Life sciences [x] Behavioural & social sciences [ ] Ecological, evolutionary & environmental sciences

For a reference copy of the document with all sections, see nature.com/documents/nr-reporting-summary-flat.pdf

Behavioural & social sciences study design

All studies must disclose on these points even when the disclosure is negative.

Study description	This is a quantitative study linking together publicly available secondary datasets to form a cross-country panel of 33 middle incoem countries, which is used to test whether increases in food prices predict increases or decreases in poverty rates at the $3.20/day poverty line, conditional upon a countries urban population share.
Research sample	We use national data for a sample of 33 middle income countries, including lower middle and upper middle income countries. The sample is not represenative of all middle income countries; selection of the countries was determined by data availability.
Sampling strategy	We first accessed the World Bank's Poverty and Inequality Platform (PIP) database to identify countries that conducted surveys on an annual basis. We then merged these data with IMF and FAO data on the consumer food price index, the total price index and the share of food in the CPI, which were used to construct a measure of the food/nonfood CPI ratio, a measure of real food price changes. This resulted in a sample of 33 middle income countries. Additional control variables were then added from the World Bank's World Development Indicators. Finally, FAO production indices were added in order to explore the association between food prices changes and agricultural production growth. Together, these 7 data sets were sufficient to explore the associations between real food prices changes and poverty changes, and food price changes and agricultural production changes, controlling for potential confounding factors.
Data collection	We downloaded secondary datasets from the websites described above as Microsoft Excel files. These were converted to Stata v17 data files and then merged with each other using country identifiers and years using Stata v17. For some countries we imputed food weights in the consumer price index. The study was non-experimental in nature.
Timing	The main food price datasets we use only cover the year 2000 onwards, and the World Bank poverty estimates end in 2019. Our final dataset is therefore an unbalanced panel of 33 countries over the period 2000-2019.
Data exclusions	Poverty estimates data were excluded if they were not reported on an annual basis.
Non-participation	This is not applicable to this study as it used secondary datasets.
Randomization	Randomization was not applicable as this is a cross-country panel data analysis that is ecological in nature. The study was therefore observational rather than experimental.

Reporting for specific materials, systems and methods

We require information from authors about some types of materials, experimental systems and methods used in many studies. Here, indicate whether each material, system or method listed is relevant to your study. If you are not sure if a list item applies to your research, read the appropriate section before selecting a response.

Materials & experimental systems

n/a	Involved in the study	
[x]	[ ]	Antibodies
[x]	[ ]	Eukaryotic cell lines
[x]	[ ]	Palaeontology and archaeology
[x]	[ ]	Animals and other organisms
[x]	[ ]	Human research participants
[x]	[ ]	Clinical data
[x]	[ ]	Dual use research of concern

Methods

n/a	Involved in the study	
[x]	[ ]	ChIP-seq
[x]	[ ]	Flow cytometry
[x]	[ ]	MRI-based neuroimaging
